# Supplementary material for: Potential Mechanisms Underlying Hypoxia-Induced Diabetes in a Rodent Model: Implications for COVID-19
Source: Children (Basel). 2021 Dec 14;8(12):1178. doi: 10.3390/children8121178 (PMC8700366; doi:10.3390/children8121178)

children

MDPI

Supplementary Materials:

Figure S1 Western blot analysis results of KCC2 and β-actin proteins harvested from primary islets in Figure 1c.

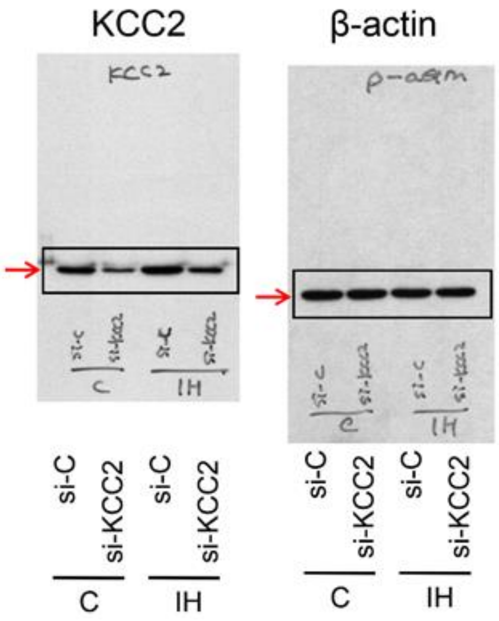

Figure S2 Western blot assay results of KCC2 protein levels in control and IH-exposed islets treated with inhibitor (VU) and vehicle (-) in Figure 2c

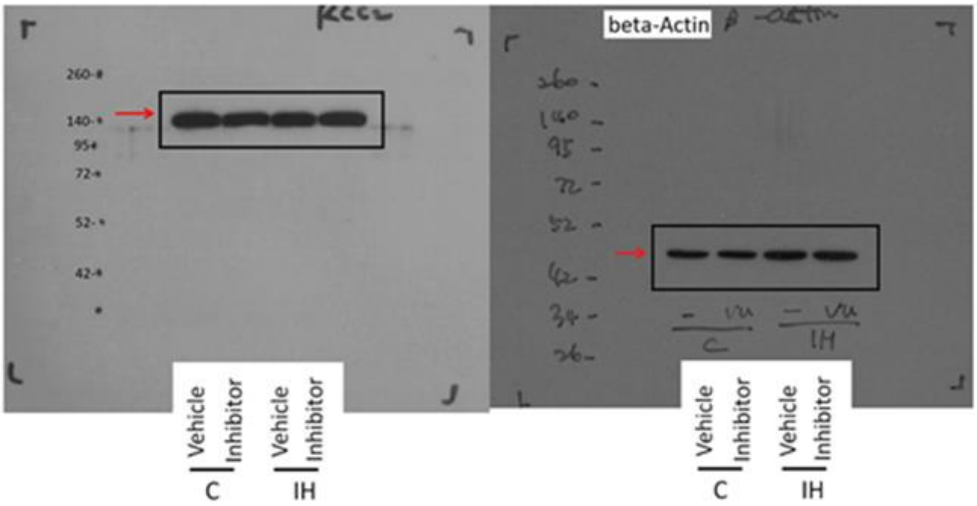

Figure S3-1 Western blot results of K_{ATP} channel-associated proteins and KCC2 protein in Figure 3b.

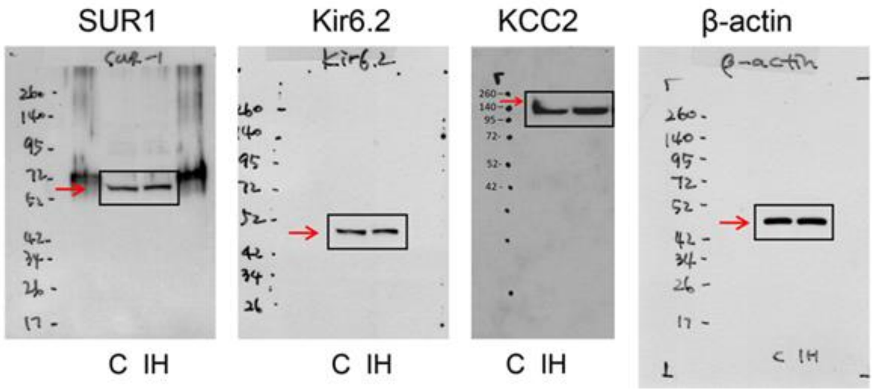

Figure S3-2 Western blot results of NKCC1, KCC2 and Pan-Cad proteins harvested from IH-treated and control animals in Figure 3c.

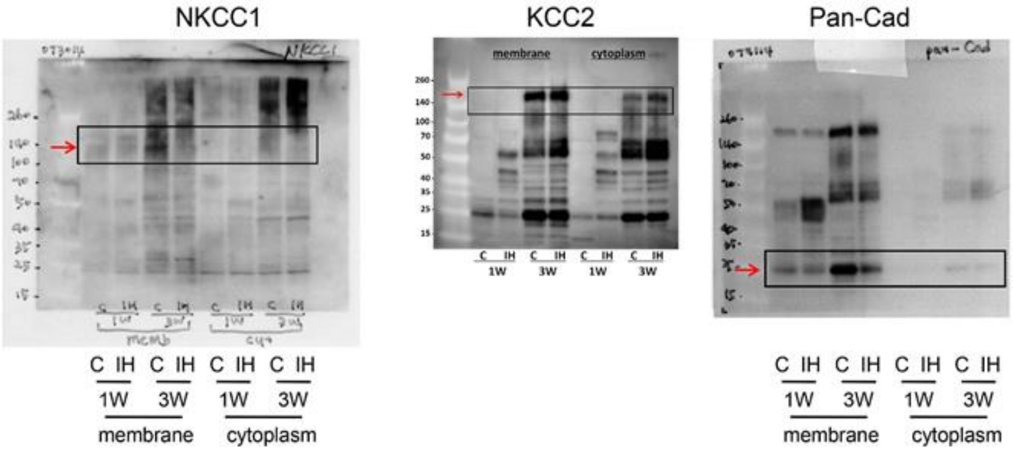

Supplement: Supplementary file 1 [file children-08-01178-s001.zip › children-1482927-supplementary.pdf]
